# Assessing sigmoidal function on memristive maps

**DOI:** 10.1016/j.heliyon.2024.e27781

**Published:** 2024-03-13

**Authors:** Vo Phu Thoai, Viet-Thanh Pham, Giuseppe Grassi, Shaher Momani

**Affiliations:** a Faculty of Electrical and Electronics Engineering, Ton Duc Thang University, Ho Chi Minh City, Viet Nam; b Department of Engineering for Innovation, University of Salento, 73100 Lecce, Italy; c Nonlinear Dynamics Research Center (NDRC), Ajman University, Ajman 20550, United Arab Emirates; d Department of Mathematics, Faculty of Science, University of Jordan, Amman 11942, Jordan

**Keywords:** Dynamics, Symmetry, Nonlinear, Chaos

## Abstract

Memristors offer a crucial element for constructing discrete maps that have garnered significant attention in complex dynamics and various potential applications. In this study, we have integrated memristive and sigmoidal function to propose innovative mapping techniques. Our research confirms that the amalgamation of memristor and sigmoidal functions represents a promising approach for creating both 2D and 3D maps. Particularly noteworthy are the chaotic maps featuring multiple sigmoidal functions and multiple memristors, as highlighted in our findings. Specifically focusing on the novel STMM_1_ map, we delve into its dynamics and assess its feasibility. Intriguingly, the introduction of sigmoidal functions leads to alterations in the quantity of fixed points and the symmetry of the map.

## 1 Introduction

The distinctive feature of a memristor, characterized by its pinched hysteresis loop, sets it apart from classical resistors. Unlike conventional resistors, a memristor exhibits a dynamic current-voltage relationship that offers inherent memory capabilities [1]. This unique attribute opens new frontiers for the development of advanced applications, particularly in the realms of neuromorphic systems and memories [2], [3], [4]. The advantages presented by memristors significantly enhance the feasibility of memristive systems, enabling them to operate reliably while consuming low power [5], [6]. Moreover, the memristor's utility extends beyond conventional roles; it serves as a nonlinear element in constructing chaotic systems [7]. Interestingly, Lai et al. have invented memristive neural networks [8] and grid-scroll attractors [9]. These systems have found diverse applications in encryption and secure communications, leveraging the inherent properties of memristors for robust and sophisticated data security [10], [11].

Unlike continuous systems, discrete systems are structured using discrete time steps, offering a different approach to modeling dynamic processes [12], [13], [14], [15]. The utility of discrete maps as a prominent tool for investigating dynamical systems spans across a multitude of disciplines, encompassing fields from nature and physics to engineering. Even seemingly simple discrete maps, such as the logistics map or Lozi map, have proven to exhibit chaotic behavior. This has led to a myriad of publications presenting diverse collections of chaotic maps [16], [17]. Exploration into various types of maps has unveiled their chaotic properties, sparking their application in numerous crucial tasks like surveillance missions, signal generation, and ensuring security measures [18], [19], [20]. Nonlinear components are at the heart of generating these discrete maps, serving as the main elements that drive and shape their dynamic behavior. The interplay of these components contributes significantly to the richness and complexity of discrete maps, allowing for their multifaceted applications across a wide spectrum of scientific and practical domains [21], [22], [23].

In recent times, there has been a rapid surge in interest surrounding memristive maps, particularly following the discovery of hidden attractors [24], [25], [26]. Memristive maps can be effectively implemented using hardware such as microcontrollers, DSP, and FPGAs, facilitating straightforward integration and fostering their suitability for various applications [27], [28]. Hyperchaotic map is reported in [29] while fractional order map is studied in [30]. Bao et al. have used four discrete memristors to develop maps [31]. The prevalence of symmetry within attractors is a common observation in memristive maps, drawing attention due to their distinctive dynamics and well-defined structures. These unique characteristics have captivated researchers, prompting ongoing endeavors to develop more effective methodologies for designing and unveiling new maps [32], [33].

The primary objective of this paper is to advance the field of memristive maps by introducing an innovative approach to create novel memristive systems. In Section 2, we present our novel approach, integrating a sigmoidal function with a memristor to formulate a discrete map. Section 3 focuses on a detailed exploration of a specific example, the STMM_1_ map, elucidating its dynamics comprehensively. Section 4 consolidates discussions on extending our approach to higher-order maps. Finally, the concluding section encapsulates the key findings and contributions of our work.

## 2 Sigmoidal function in maps

**Figure 1:**
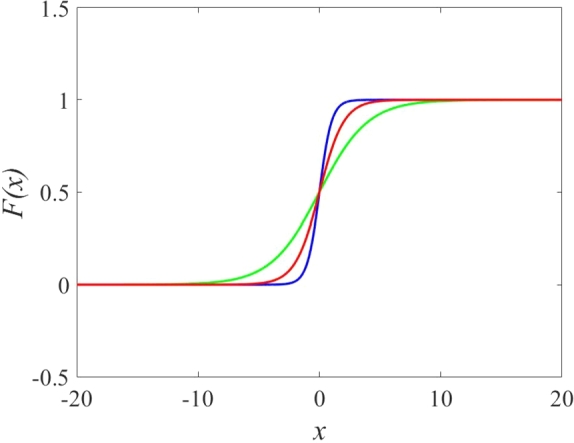
Sigmoidal curves for: *b* = 1 (red), *b* = 0.5 (green), and *b* = 2 (blue).

The sigmoidal function is integral across various domains due to its nonlinear nature and smooth characteristics. Formula (1) defines a sigmoidal function exhibiting an S-shaped curve, as depicted in Fig. 1.

(1) $$F\left(x\right)=\frac{1}{1+e^{-bx}}.$$

**Figure 2:**
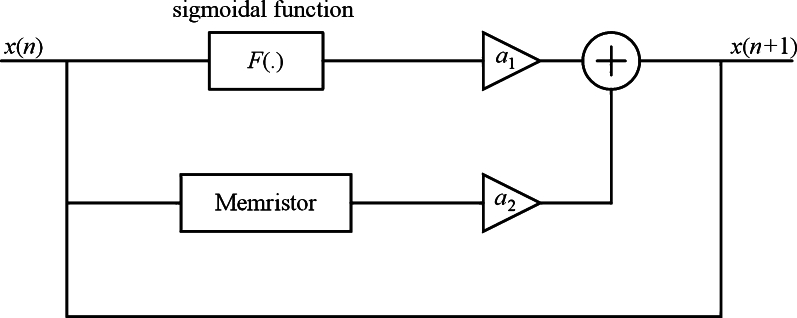
Map's diagram including a sigmoidal function and a memristor.

Fig. 1 illustrates how the sigmoidal function efficiently transforms an input *x* into a confined range of outputs. Consequently, leveraging the sigmoidal function proves highly advantageous in the development of artificial neural networks. In Fig. 2, we present a discrete map diagram resulting from the integration of a sigmoidal function and a memristor. Parameters *a* and *b* delineate the influence of the sigmoidal and memristive components, respectively.

Based on Fig. 2, map's model is

(2) $$\begin{array}{c} x\left(n+1\right)=\frac{a_{1}}{1+e^{-bx(n)}}+a_{2}x\left(n\right)M\left(y\left(n\right)\right),\\ y\left(n+1\right)=y\left(n\right)+x\left(n\right), \end{array}$$

with discrete memristance M(y(n)). By solving (3)

(3) $$\begin{array}{c} x^{⁎}=\frac{a_{1}}{1+e^{-bx^{⁎}}}+a_{2}x^{⁎}M\left(y^{⁎}\right),\\ y^{⁎}=y^{⁎}+x^{⁎}, \end{array}$$

fixed points $\left(x^{⁎},y^{⁎}\right)$ must satisfy the condition (4)

(4) $$\begin{array}{c} \frac{a_{1}}{1+e^{-bx^{⁎}}}=0,\\ x^{⁎}=0. \end{array}$$

For $a_{1}\neq 0$, map (2) has no fixed point.

Interestingly, specific chaotic maps can be derived from (2). Let take $M\left(y\left(n\right)\right)={\left(y\left(n\right)\right)}^{2}-1$ [31], we obtain sigmoid-term memristive map (STMM_1_) given by (5):

(5) $$\begin{array}{c} x\left(n+1\right)=\frac{a_{1}}{1+e^{-bx\left(n\right)}}+a_{2}x\left(n\right)\left({\left(y\left(n\right)\right)}^{2}-1\right)\\ y\left(n+1\right)=y\left(n\right)+x\left(n\right) \end{array}$$

**Figure 3:**
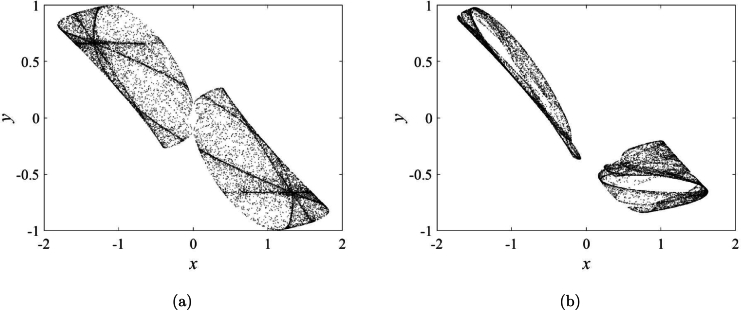
(**a**) Symmetry (*a*_1_ = 0), and (**b**) asymmetry (*a*_1_ = 0.25) in iterative plot.

Fig. 3a displays chaos and symmetry in iterative plot with parameters (6)

(6) $$\begin{array}{c} a_{1}=0\\ a_{2}=1.78\\ \left(x(0),y\left(0\right)\right)=\left(-0.5,0.5\right) \end{array}$$

Symmetry is often observed in memristive maps. However, when changing $a_{1}$, STMM_1_ map exhibits asymmetry (see Fig. 3b) for parameters (7):

(7) $$\begin{array}{c} a_{1}=0.25\\ a_{2}=1.78\\ \left(x(0),y\left(0\right)\right)=\left(-0.5,0.5\right) \end{array}$$

In two cases, the maximum Lyapunov exponents are 0.2364 and 0.2885. Dynamics of STMM_1_ map is considered more detail in the next section. Our designed map reveals an uncommon asymmetric attractor, a rarity among existing memristive maps. The sigmoidal function can serve as an effective coupling component for linking various STMM maps. Consequently, leveraging STMM maps simplifies the construction of networks.

## 3 Study of STMM_1_

**Figure 4:**
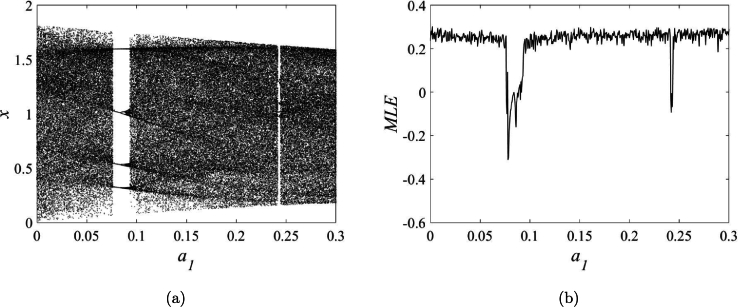
**a**) Bifurcation diagram, (**b**) maximum Lyapunov exponents for $a_{1}\in \left[0,0.3\right]$, *a*_2_ = 1.78, and (x(0),y(0))=(−0.5,0.5).

Unlike classical discrete maps characterized by fixed points, the stability assessment of fixed points in STMM_1_ map for $a_{1}\neq 0$ cannot be determined. Dynamics of STMM_1_ are shown in Fig. 4a and Fig. 4b via bifurcation diagram and maximum Lyapunov exponents for $a_{1}$. Chaotic and non-chaotic behaviors are observed for $a_{1}\in \left[0,0.3\right]$. Non-chaotic behavior can be found in the ranges (0.076,0.093) and (0.241,0.244).

**Figure 5:**
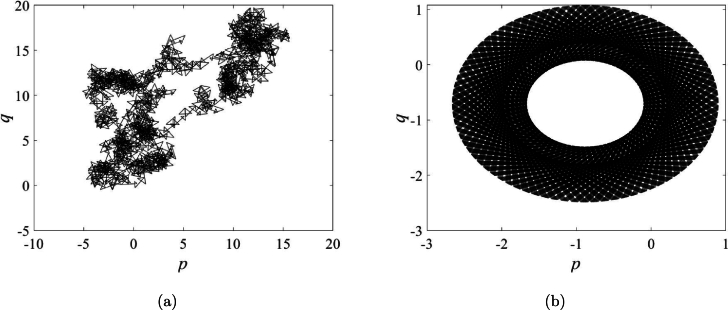
The 0–1 test: (**a**) *a*_1_ = 0.25, (**b**) *a*_1_ = 0.08.

The 0-1 test has been utilized to verify the presence of chaos within the map, serving as a visual tool for confirmation. Chaotic behavior is illustrated in Fig. 5a, displaying patterns reminiscent of Brownian motion. Conversely, Fig. 5b exhibits periodic traits observed in trajectories constrained within specific bounds.

**Figure 6:**
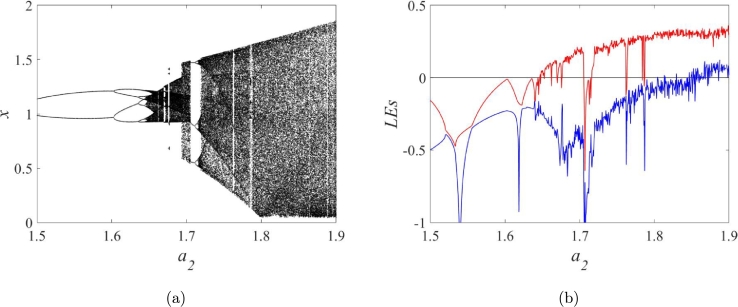
**a**) Bifurcation diagram, (**b**) Lyapunov exponents for $a_{2}\in \left[1.5,1.9\right]$, *a*_1_ = 0.25, and (x(0),y(0))=(−0.5,0.5).

**Figure 7:**
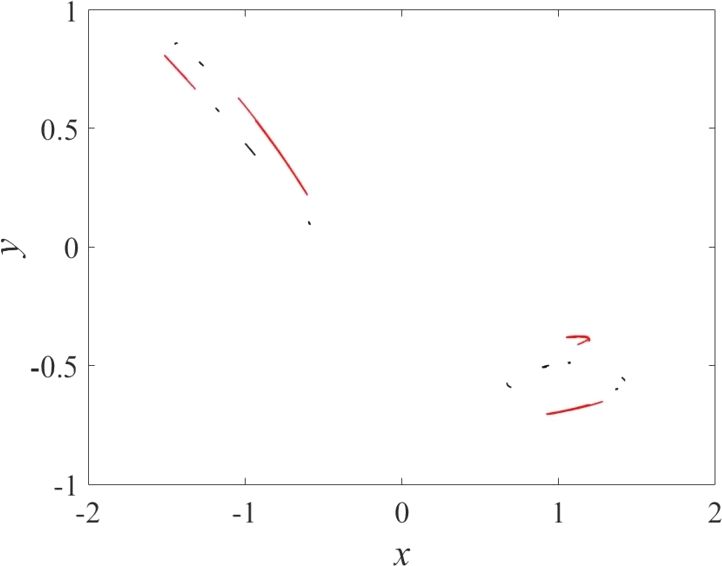
Multistability for *a*_1_ = 0.25, *a*_2_ = 1.677 and (x(0),y(0))=(−0.5,0.5) (black), (x(0),y(0))=(−0.1,0.1) (red).

When changing $a_{2}$ (see Fig. 6a and Fig. 6b), the map exhibits non-chaotic, chaotic, and hyperchaotic behaviors. Specially, multistability can be observed in STMM_1_ map. As illustrated in Fig. 7, two attractors coexist for different initial conditions with the same parameters $a_{1}=0.25$, $a_{2}=1.677$.

**Figure 8:**
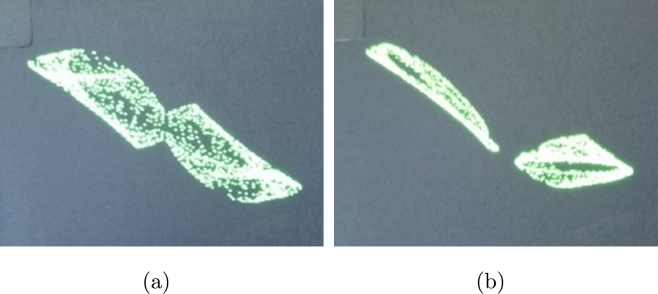
(**a**) Symmetry (*a*_1_ = 0), and (**b**) asymmetry (*a*_1_ = 0.25) captured from the board.

The feasibility of STMM_1_ map was explored through its hardware execution. To achieve this, an Arduino Uno board was selected for its simplicity, constrained resources, and cost-effectiveness. Additionally, an accessible debugging and display tool, the Serial Plotter, facilitated the process. The equation was programmed in the Arduino Integrated Development Environment (IDE) and uploaded onto the board. The experimental outcomes vividly demonstrate the emergence of chaos as depicted in Fig. 8a and Fig. 8b. Complex dynamics and feasibility of STMM_1_ map are suitable for lightweight ciphers specifically tailored for Internet of Things (IoT) applications.

## 4 Discussion

As detailed in Section 2, model (2) facilitates the creation of diverse memristor maps by adjusting discrete memristance. Furthermore, this section demonstrates the methodology for crafting high-order maps through the utilization of sigmoidal functions. Our focus within this section is on two distinct classes of high-order maps: those featuring multiple sigmoidal functions and those incorporating multiple memristors.

### 4.1 Map with multiple sigmoidal functions

**Figure 9:**
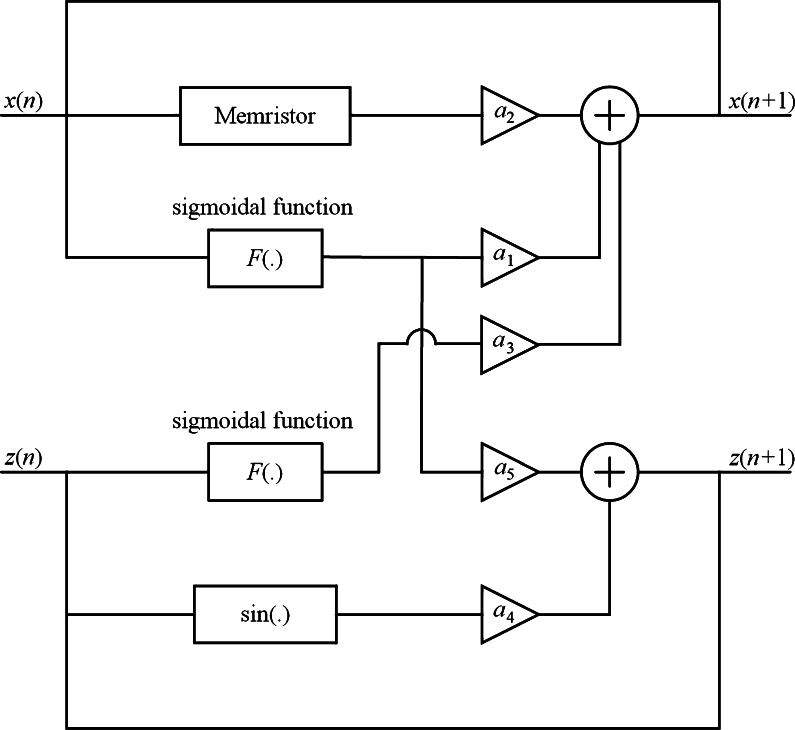
Using sigmoidal functions and a memristor to design new maps.

Structure of maps with multiple sigmoidal functions is proposed in Fig. 9. Mathematical model is given by (8):

(8) $$\begin{array}{c} x\left(n+1\right)=\frac{a_{1}}{1+e^{-bx(n)}}+a_{2}x\left(n\right)M\left(y\left(n\right)\right)+\frac{a_{3}}{1+e^{-bz(n)}},\\ y\left(n+1\right)=y\left(n\right)+x\left(n\right),\\ z\left(n+1\right)=a_{4}\sin\left(z\left(n\right)\right)+\frac{a_{5}}{1+e^{-bx(n)}}, \end{array}$$

where $a_{i}$ are parameters.

With discrete memristance (9) [31]

(9) $$M\left(y\left(n\right)\right)={\left(y\left(n\right)\right)}^{2}-1,$$

we get the memristive map (named STMM_2_ map) given by (10):

(10) $$\begin{array}{c} x\left(n+1\right)=\frac{a_{1}}{1+e^{-bx\left(n\right)}}+a_{2}x\left(n\right)\left({\left(y\left(n\right)\right)}^{2}-1\right)+\frac{a_{3}}{1+e^{-bz\left(n\right)}},\\ y\left(n+1\right)=y\left(n\right)+x\left(n\right)\\ z\left(n+1\right)=a_{4}\sin\left(z\left(n\right)\right)+\frac{a_{5}}{1+e^{-bx(n)}}. \end{array}$$

**Figure 10:**
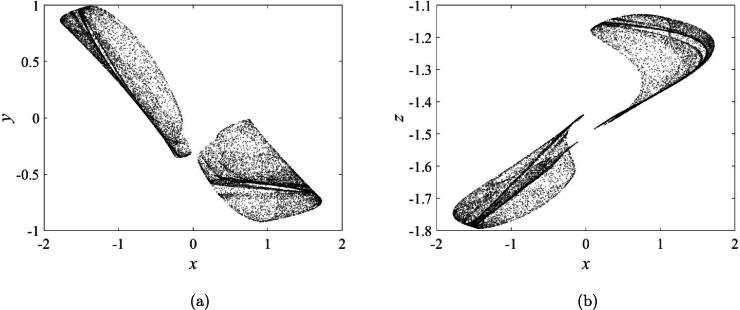
Iterative plot in (**a**) *x* − *y*, and (**b**) *x* − *z*.

STMM_2_ map generates chaos (see Fig. 10a and Fig. 10b) with parameters (11)

(11) $$\begin{array}{c} \left(x\left(0\right),y\left(0\right),z\left(0\right)\right)=\left(0.01,0.01,-0.1\right),\\ a_{1}=0.1,\\ b=1,\\ a_{2}=1.78,\\ a_{3}=0.1,\\ a_{4}=1,\\ a_{5}=-1. \end{array}$$

### 4.2 Map with multiple memristors

**Figure 11:**
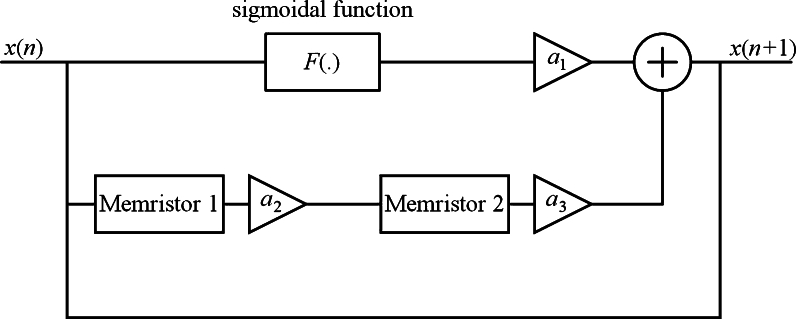
Structure of map including a sigmoidal function and two memristors.

To construct a category of maps involving multiple memristors, we introduced the structural framework illustrated in Fig. 11. This configuration comprises a sigmoidal function integrated with two memristors. The mathematical model derived from Fig. 11 is represented by (12):

(12) $$\begin{array}{c} x\left(n+1\right)=\frac{a_{1}}{1+e^{-bx\left(n\right)}}+a_{2}a_{3}x\left(n\right)M_{1}\left(y\left(n\right)\right)M_{2}\left(z\left(n\right)\right),\\ y\left(n+1\right)=y\left(n\right)+x\left(n\right),\\ z\left(n+1\right)=z\left(n\right)+a_{2}x\left(n\right)M_{1}\left(y\left(n\right)\right), \end{array}$$

with parameters $a_{i}$.

With discrete memristances (13) and (14) [31]

(13) $$M_{1}\left(y\left(n\right)\right)=c\left|y\left(n\right)\right|-1,$$

and

(14) $$M_{2}\left(z\left(n\right)\right)={\left(z\left(n\right)\right)}^{2}-1,$$

we get the memristive map (STMM_3_ map) described as (15):

(15) $$\begin{array}{c} x\left(n+1\right)=\frac{a_{1}}{1+e^{-bx\left(n\right)}}+a_{2}a_{3}x\left(n\right)\left(c\left|y\left(n\right)\right|-1\right)\left({\left(z\left(n\right)\right)}^{2}-1\right),\\ y\left(n+1\right)=y\left(n\right)+x\left(n\right),\\ z\left(n+1\right)=z\left(n\right)+a_{2}x\left(n\right)\left(c\left|y\left(n\right)\right|-1\right). \end{array}$$

For the parameter values (16)

(16) $$\begin{array}{c} \left(x\left(0\right),y\left(0\right),z\left(0\right)\right)=\left(0.01,0.01,0.01\right),\\ b=1,\\ a_{2}=-1.7,\\ a_{3}=1.15,\\ c=0.35. \end{array}$$

**Figure 12:**
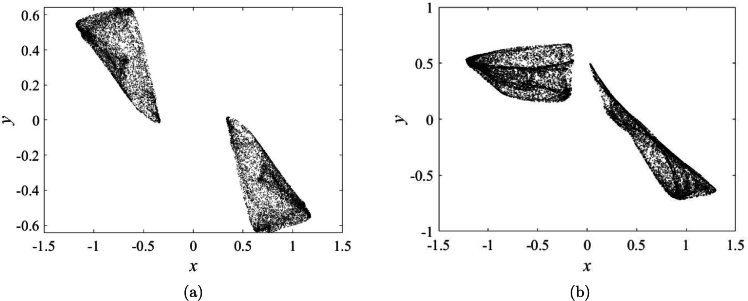
Iterative plot for (**a**) *a*_1_ = 0, and (**b**) *a*_1_ = −0.2.

STMM_3_ map exhibits chaos as displayed in Fig. 12a and Fig. 12b. Both symmetry and asymmetry can be seen in iterative plots.

## 5 Conclusions

This paper introduces a novel approach to designing a chaotic map using both sigmoidal and memristive components. Our primary focus has been on the examination of a specific map termed as the STMM_1_ map, which exhibits compelling traits such as chaos, asymmetry, and the absence of fixed points. We delve into the dynamics and realization of this map in detail. Additionally, through the extension of this approach, we demonstrate the construction of high-order memristive maps. To showcase the efficacy of this extension, we introduce two categories of maps: one incorporating multiple sigmoidal functions and another amalgamating multiple memristive components. The potential application of such memristive maps in lightweight ciphers for real-world IoT will be assessed in coming researches.

## CRediT authorship contribution statement

**Vo Phu Thoai:** Software, Methodology, Conceptualization. **Viet-Thanh Pham:** Writing – original draft, Resources, Investigation. **Giuseppe Grassi:** Visualization, Methodology, Funding acquisition. **Shaher Momani:** Writing – review & editing, Validation, Supervision.

## Declaration of Competing Interest

The authors declare that they have no known competing financial interests or personal relationships that could have appeared to influence the work reported in this paper.

## Data Availability

No data was used for the research described in the article.
